# T-Plastin Expression Downstream to the Calcineurin/NFAT Pathway Is Involved in Keratinocyte Migration

**DOI:** 10.1371/journal.pone.0104700

**Published:** 2014-09-16

**Authors:** Cécilia Brun, Agathe Demeaux, Frédéric Guaddachi, Francette Jean-Louis, Thierry Oddos, Martine Bagot, Armand Bensussan, Sébastien Jauliac, Laurence Michel

**Affiliations:** 1 INSERM U976, Centre de Recherche sur la Peau, Hôpital Saint Louis, Paris, France, and Université Paris Diderot, Sorbonne Paris cité, Paris, France; 2 Johnson & Johnson Santé Beauté France, Centre de Recherche, Val de Reuil, France; University of Tennessee, United States of America

## Abstract

Cutaneous wound healing requires keratinocyte proliferation, migration and differentiation to restore the barrier function of the skin. The calcineurin/nuclear factor of activated-T-cell (NFAT) signaling pathway has been recently shown to be involved in keratinocyte growth, differentiation and migration. It is induced by an increased intracellular calcium rate and its inhibition results in decreased capacities of keratinocytes to migrate. Nevertheless, the link between calcineurin activation and keratinocyte migration remains unknown. Recently, Orai1, a pore subunit of a store-operated calcium channel that favors calcium influx, was shown to play a critical role to control proliferation and migration of basal keratinocytes. Of interest, the actin-bundling T-plastin is crucial in cell motility through cross-linking to actin filament and its synthesis was shown to be induced by calcium influx and regulated by the calcineurin/NFAT pathway in tumor Sezary cells. We investigated herein the role of the calcineurin/NFAT pathway-dependent T-plastin in keratinocyte migration, by quantifying T-plastin expression in keratinocytes and by analyzing their migration under calcineurin inhibition or knockdown of NFAT2 or T-plastin. We did confirm the role of the calcineurin/NFAT pathway in keratinocyte migration as shown by their decreased capacities to migrate after FK506 treatment or siNFAT2 transfection in both scratching and Boyden assays. The expression of NFAT2 and T-plastin in keratinocytes was decreased under FK506 treatment, suggesting that T-plastin plays a role in keratinocyte migration downstream to the calcineurin/NFAT pathway. Accordingly, siRNA knockdown of T-plastin expression also decreased their migration capacities. Actin lamellipodia formation as well as FAK and β6-integrin expression were also significantly decreased after treatment with FK506 or siRNA, reinforcing that NFAT2-dependent T-plastin expression plays a role in keratinocyte migration. These results indicate that T-plastin might be considered as a major actor in the mechanisms underlying calcineurin/NFAT-dependent keratinocyte migration and may explain wound-healing defects observed in patients under calcineurin inhibitor long-term treatment.

## Introduction

The calcineurin/nuclear factor of activated T cell (NFAT) signaling pathway is a major regulator of cell motility in several cell types [1], [2]. This cascade is induced by an increase rate of intracellular calcium, which binds to calmodulin, leading to activation of the calcium-sensitive phosphatase calcineurin and allowing NFAT dephosphorylation and nuclear translocation with consequent transcriptional activation of several genes. Recently, the calcineurin/NFAT signaling pathway has been shown to be involved in keratinocyte growth and differentiation control [3] as well as in their migration following activation by lysophosphatidic acid (LPA) [4]. Indeed, LPA induces a mobilization of calcium through the calcium sensor STIM-1 and the membrane ion channel ORAI-1, leading to the activation of the calcineurin/NFAT pathway. Inhibition of this signaling cascade by cyclosporine A (CspA) or using NFAT2 (NFATc1) siRNA resulted in a decrease in keratinocyte capacities to migrate. A very recent study showed that ORAI-1 channel plays a prominent role in supplying calcium enable to drive migration of keratinocytes through Focal Adhesion Kinase (FAK) turnover [5]. Nevertheless, the link between calcium–induced calcineurin activation and keratinocyte migration remains unknown.

T-plastin is one of the three forms of the actin-bundling protein, which belongs to the fimbrin family [6]. We recently showed that T-plastin synthesis could be induced by calcium entry and regulated by the calcineurin/NFAT pathway in tumor T cells from patients with Sezary Syndrome [7]. Specific T-plastin upregulation favored tumor T cell migration that was downregulated by calcineurin inhibitors, including CspA or FK506 (Tacrolimus). T-plastin is involved in cell motility through its cross-linking to actin filament, helping to the assembly and stabilization of the actin network [8]. This suggests that T-plastin might be a candidate that favors calcineurin/NFAT-dependent keratinocyte migration.

The aim of the present work was to assess the role of T-plastin-dependent calcineurin pathway in keratinocyte migration, by quantifying T-plastin expression in both primary keratinocytes and NCTC cell line and by studying cell migration with or without exposure to Tacrolimus or after transfection with siRNA for NFAT2 or T-plastin.

## Materials and Methods

### Keratinocyte extraction and cell culture

Primary keratinocyte cultures were obtained as described previously [9] from surgical residues of normal skin obtained from healthy volunteers undergoing plastic surgery after written informed consent of non-opposition were signed. All the data concerning the healthy volunteers were anonymized upon collection of surgical residues by surgeons prior to culturing. The authors did not at all participate in the collection of this tissue and just got anonymous surgical residues. None of the authors have access to identifying information about donors of skin surgical residues. Briefly, slices of skin samples were incubated with 0.25% trypsin to allow epidermis detachment. Obtained single-cell suspensions of epidermal cells were cultured in Dulbecco's modified Eagle's medium (DMEM, Gibco) supplemented with 10% fetal bovine serum (FBS), 100 U/ml penicillin, 100 mg/ml streptomycin (DMEM complete medium) in a 90% humidified incubator with 5% CO2 at 37°C. After adhesion of keratinocytes, the medium was supplemented with epidermal growth factor, cholera toxin and hydrocortisone.

The NCTC 2544 cell line, kindly provided by Pr Gottfried Leonhardi to our laboratory [10], was cultured in DMEM complete medium and was especially used for cell transfection.

### Cell Transfection

Transient transfection of NCTC cells was carried out using Lipofectamine 2000 Transfection Reagent (Life Technologies) according to the manufacturer's protocol. For siRNA transfection, cells were incubated with 60 nM of siRNA pool targeting either T-plastin or NFAT2 (Thermoscientific) for 24 hours before scratching assays or RNA and protein extraction. As control siRNA, a non-relevant siRNA sequence pool (Thermoscientific) was used.

In order to assess NFAT transcriptional activity, cells were transfected with PGL4 Luciferase Reporter Vector containing NFAT response element (NFAT-Luc vector, Promega) (2 µg) in association or not with siRNA and were co-transfection with β-galactosidase reporter vector (pSV-β-Galactosidase Control Vector, Promega) (0.2 µg) for normalisation of luciferase activity. In some cases, cells were pre-treated with FK506 for 30 minutes. Twenty four hours after transfection, cells (1.10^6^) were harvested by trypsinisation, washed twice with PBS, and lysed with Reporter Lysis Buffer before luciferase assay detection (Luciferase Assay System, Promega) and assessment of β-galactosidase activity using the β-Galactosidase Enzyme Assay System (Promega). All the assays were performed in duplicate.

### RNA extraction, Reverse transcription and Quantitative PCR

Expression of several mRNA was analyzed by real-time quantitative PCR (qRT-PCR) using the 7300 System SDS Software (Applied Biosystems). Total RNA was isolated from 10^6^ cells with RNeasy extraction kit (Qiagen) according to the manufacturer's instructions. RNAs were reverse-transcripted with a Thermoscript RT-PCR system kit (Invitrogen). Primers were obtained from Eurogentec. Q-PCR reactions were performed with the Power SYBR Green PCR master mix in a MicroAmp optical 96-well reaction plate according to the manufacturer's instructions (Applied Biosystems). Gene expression levels were normalized by β2-microglobulin expression and expressed as 2-ΔCT (Arbitrary Units) as previously described [7].

### Western Blotting

Cells (2.10^6^) were lysed for 20 minutes at 4°C in RIPA lysis buffer (Rockland Immunochemicals) supplemented with protease inhibitor cocktail (Sigma-Aldrich). After centrifugation (14 000 rpm, 15 minutes, 4°C), the concentration of the protein extracts was evaluated by a Bradford assay and 100 µg of protein extract were loaded onto 8% polyacryl-amide gels containing sodium dodecyl sulfate (SDS), subjected to electrophoresis, and transferred to nitrocellulose membrane. The blot was blocked with 5% nonfat milk for 1 hour and incubated overnight at 4°C with specific primary antibodies. They were subsequentially exposed to anti–mouse (1/3000) or anti-rabbit (1/10000) antibody coupled to horseradish peroxidase for 1 hour. An enhanced chemiluminescent system was used for detection on the ImageQuant system. Equal protein loading was confirmed by the use of monoclonal GAPDH antibody (Sigma-Aldrich).

### Scratch wound healing assay

Wound healing assay was conducted using 35 mm culture dishes. When confluency was reached, the cells were wounded with a plastic pipette tip. Photographs were taken at scratching (day 0) and after 24 h (day 1). Measurement of the distance between the wound margins was assessed on 10 photographs per condition and the percentage of closure was calculated as the ratio of the mean distances measured at day 1 relative to the ones obtained at day 0.

### Transwell Migration Assay

Cell migration was assessed quantitatively with a Transwell chamber. Medium with 0.5% FBS or 10% FBS (with or without 10 µM of FK506) was added to the bottom of a 24-well companion plate (BD Falcon), and an insert with a 8-µm pore-size PET membrane (BD Falcon) was placed over the wells. Subconfluent cells pretreated or not with 10 µM FK506 were trypsinized and resuspended in DMEM with 0.5% FBS at 10^6^ cells/ml. A 100 µl aliquot of the keratinocyte suspension was added to the upper wells, and the chamber was incubated for 24 hours at 37°C, 5% CO2. The cells of the upper surface of the membrane were removed by scraping with a cotton swab. The migrated cells on the lower surface of the membrane were stained with hematoxylin, were observed in microscope at x10 and the number of migrated cells was counted.

### Immunocytochemistry

Cells were fixed in 3% paraformaldehyde, washed with Phosphate Buffered Saline (PBS) containing 3% of albumin from bovine serum (BSA) and permeabilized with 0.1% Triton X100 for 20 minutes. After washing with PBS containing 3% BSA, cells were incubated with Phalloïdin-FITC (1 µg/ml) for 40 minutes or/and with T-plastin primary antibody (4 µg/ml) for 1 hour followed by Cy3-conjugated secondary goat antibody (10 µg/ml) for 30 minutes. Cells were mounted with Vectashield with DAPI and observed with Zeiss “AxioVert” Microscope.

### Statistical analysis

Statistical significance for all experiments was assessed using the two-sided Student's *t* test. Differences were considered as significant at *p*<0.05.

## Results and Discussion

### NFAT2 expression is involved in keratinocyte migration

**Figure 1:**
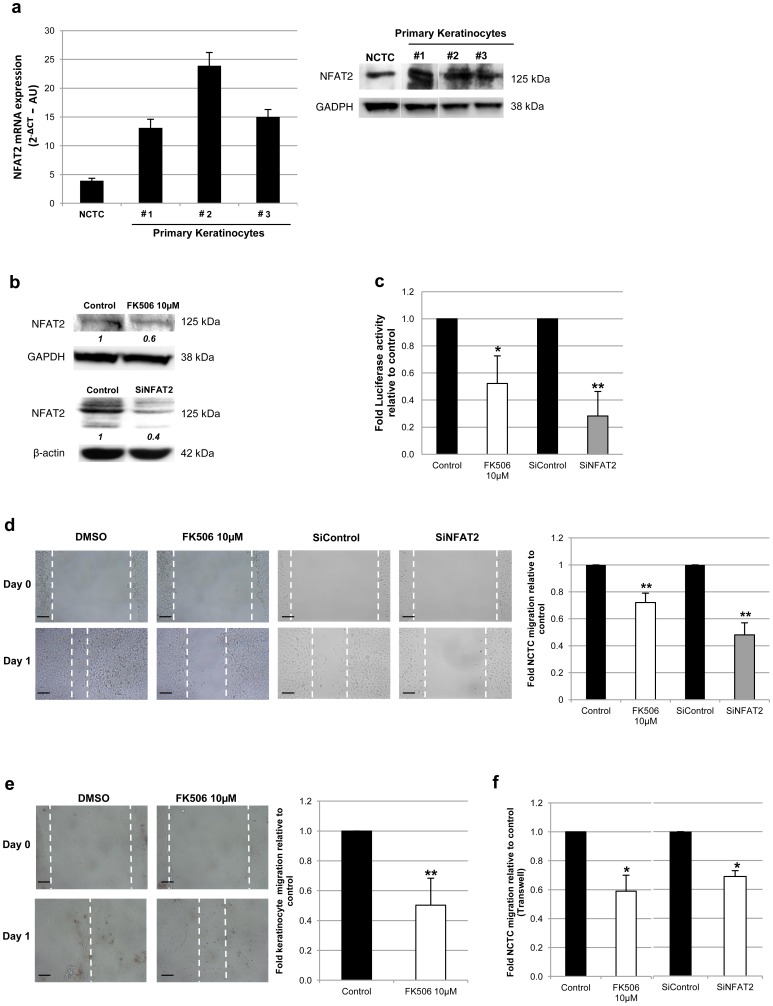
NFAT2 expression is involved in keratinocyte migration. Monolayers of confluent NCTC cells (3 independent cultures) or primary keratinocytes (n = 3) cultured during 15±3days in DMEM complete medium. (**a**) Quantification of NFAT2 mRNA or protein levels was obtained by RT-qPCR (left) and western blot (right), respectively. (**b**) NFAT2 protein expression was quantified by western blot analysis in NCTC after FK506 exposure for 24 h or after siNFAT2 transfection (60 nM). One representative blot from 3 independent experiments. The values indicated under each blot are the mean fold protein expression relative to controls taken as 1 after normalization by GAPDH or β-actin (Image J quantification). (**c**) Measurement of luciferase activity for quantification of NFAT2 transcriptional activity in NCTC cells after 24 hours of treatment with FK506 (10 µM) or transfection with siNFAT2 (60 nM). NCTC (**d**) and primary keratinocyte (**e**) cultures were treated with FK506 (10 µM) or DMSO (0.1%) or were transfected with specific pooled NFAT2 siRNA or control siRNA (60 nM) 24 hours before wounds were performed by scratching with a micropipette tip (day 0). Photographs were taken at scratching (day 0) and after 24 h (day 1), representative of one experiment (left panel of photographs). Measurement of the distance between the wound margins was assessed on 10 photographs per condition and the percentage of closure was calculated as the ratio of the mean distances measured at day 1 relative to the ones obtained at day 0 (n = 3). Dark bar  = 100 µm. (**f**) Migration of NCTC cells was carried out with a Transwell assay after 24 hours of treatment with FK506 (10 µM) or transfection with siNFAT2 (60 nM). The numbers of cells migrating through the Transwell membrane were counted and results are expressed as the ratio of the migrating cells after FK506 exposure or SiRNA transfection relative to the ones without treatment (n = 3). All results are means ± SD.*p<0.05,**p<0.01.

**Figure 2:**
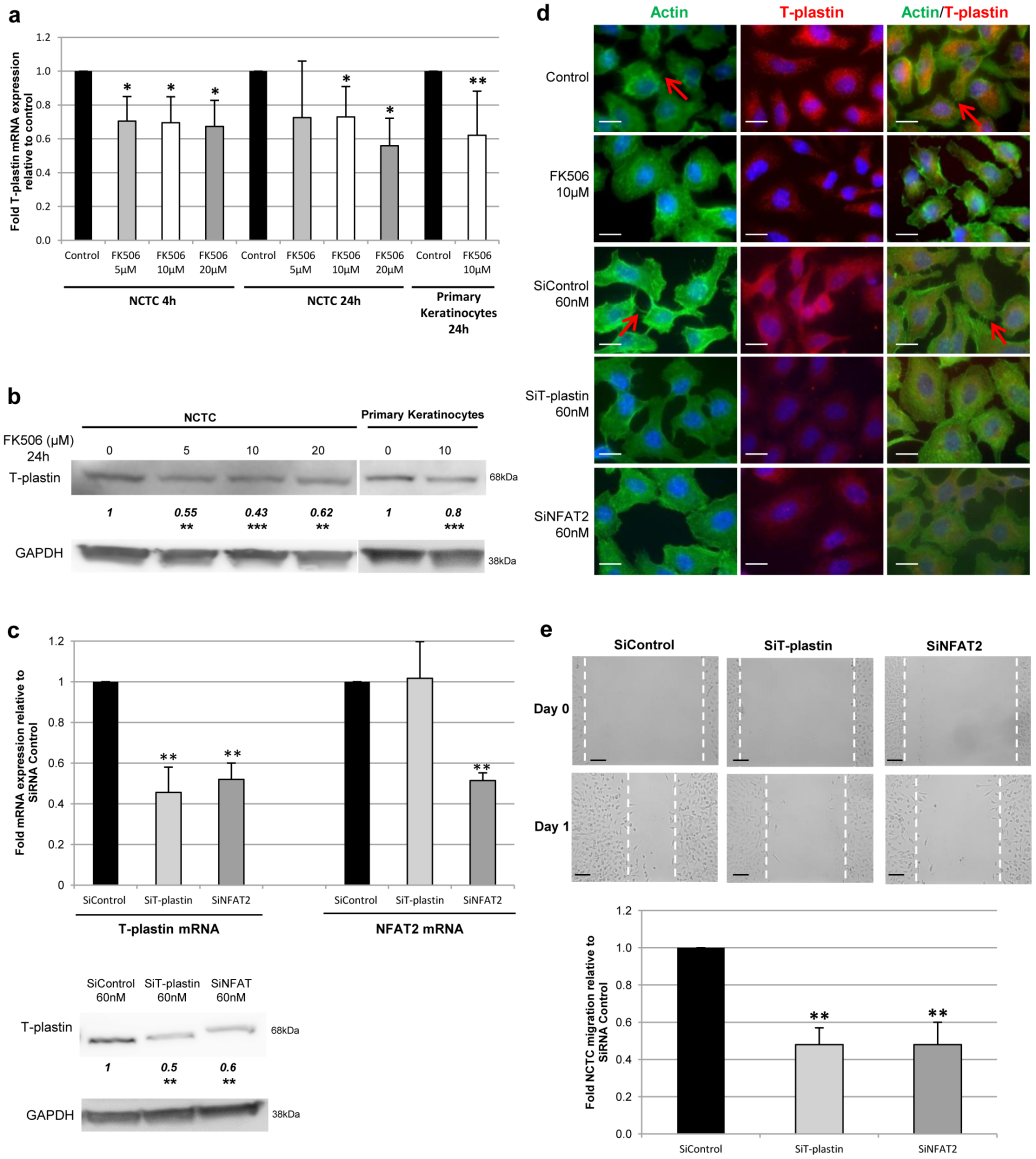
T-plastin synthesis is controlled by the NFAT pathway and is involved in keratinocyte migration. (**a**) T-plastin mRNA expression was quantified by quantitative RT-PCR in NCTC (3 independent experiments) or primary keratinocytes (n = 6) after FK506 treatment for 4 or 24 h and results are expressed as ratio relative to untreated control taken as 1. (**b**) T-plastin protein expression was quantified by western blot analysis in NCTC (3 independent experiments) or primary keratinocytes (n = 5) after FK506 exposure for 24 h. (**c**) T-plastin and NFAT2 mRNA expression (graph) and protein expression (under) were evaluated after NCTC siRNA transfection by qRT-PCR and western blot respectively (3 independent experiments). (**d**) Immunoflorescence staining of NCTC cells treated with FK506 or siRNA. Green: phalloïdine-FITC (1/100e), red: anti-T-plastin primary antibody (1/50e, Mouse) followed by Cy3-conjugated secondary antibody (1/100e, Goat). White bar  = 20 µm. (**e**) Wound closure was evaluated after transfection with specific siNFAT2 and siT-plastin *vs* siControl, as shown for one representative experiment (up) and mean inhibition relative to control (n = 3). Dark bar  = 100 µm. All results are means ± SD. The values reported under each blot are the mean fold protein expression relative to controls taken as 1 after normalization by GAPDH (Image J quantification). *p<0.05, **p<0.01, ***p<0.001.

The results of the present work did confirm the constitutive mRNA and protein expression of NFAT2 in NCTC cell line as well as in primary keratinocytes (Figure 1a). This expression was downregulated by the calcineurin inhibitor FK506 (10 µM) in both NCTC cells and keratinocytes: a 4 hour inhibition was sufficient to significantly decrease the mRNA synthesis of NFAT2 in NCTC cells by 35%±8% (3 independent experiments, *p*<0.01) whereas 24 hours of FK506 exposure were necessary in primary keratinocytes to get a 30%±16% decrease in mRNA levels (n = 6, *p*<0.01). Exposure to FK506 (10 µM) also induced a downregulation of NFAT2 protein expression, as shown in Figure 1b. This suggests that NFAT2 pathway is, at least in part, responsible for its own regulation in keratinocytes, as previously described in other cell types [11]. Furthermore, FK506 treatment did significantly inhibit NFAT2 transcriptional activity, as measured by luciferase activity using a NFAT Luc reporter assay (Figure 1c). Transfection with specific NFAT2 siRNA significantly downregulated NFAT2 expression at protein levels (Figure 1b) and at mRNA levels, as shown in Figure 2c. Moreover, NFAT2 siRNA transfection also significantly reduced NFAT2 transcriptional activity (Figure 1c).

Of interest, blocking NFAT pathway by either incubation with FK506 or downregulation of NFAT2 expression using specific siRNA resulted in a delay in wound closure induced by cell scratching of both NCTC (Figure 1d) and primary keratinocytes (Figure 1e). To evaluate whether the decrease in wound closure was due to a loss of migratory potential or to a decrease in proliferation, a MTT assay was performed for measuring proliferation of NCTC cells after 24, 48 and 72 hours of culture with FK506 concentrations ranging from 5 µM to 20 µM. Since FK506 did not reduce significantly NCTC proliferation (data not shown), our results indicate that the delayed wound closure observed after scratching in the presence of FK506 was effectively due to an impaired migration of keratinocytes. Furthermore, these results were confirmed by performing Transwell assays with FK506 as well as siNFAT2 transfection: both treatments induced a significant inhibition of the rate of migration of NCTC cells (Figure 1f).

### T-plastin synthesis is controlled by NFAT pathway and is involved in keratinocyte migration

In order to demonstrate the NFAT pathway involvement in keratinocyte T-plastin synthesis, cells were cultured in the presence of NFAT/calcineurin inhibitor or specific NFAT2 siRNA before T-plastin mRNA and protein expression was assessed. Our results show that FK506 exposure inhibited the expression of T-plastin at both mRNA (Figure 2a) and protein level (Figure 2b) in NCTC cells and in primary cultured keratinocytes. Similarly, NFAT2 knockdown by specific siRNA transfection did also reduce T-plastin expression (Figure 2c). In contrast, T-plastin synthesis was not at all modified by keratinocyte stimulation with 1 µM of ionomycin during 4 and 24 hours (data not shown). These results clearly indicate that constitutive T-plastin expression by keratinocytes is related to a sustained NFAT pathway activation *in vitro*.

Since T-plastin is known to stabilize the actin network, we studied the actin cytoskeleton of NCTC cells by using phalloïdine-FITC staining after cell exposure to FK506 or transfection with siRNA for T-plastin or NFAT2. As expected, the loss of T-plastin expression resulted in a disorganized actin network as shown by the absence of actin lamellipodia in NCTC treated with FK506 or both siRNA (Figure 2d). Keratinocyte failure to organize and stabilize the actin network through T-plastin expression could partly explain their loss of migratory capacities. Furthermore, in order to determine whether T-plastin is involved in keratinocyte migration, we downregulated its expression by using specific siRNA for T-plastin or NFAT2 in NCTC cells and then performed scratch assays. A significant delay in wound closure was observed after downregulation of NFAT2 as well as T-plastin expression, confirming a loss of migratory capacities under T-plastin or NFAT2 expression decrease (Figure 2e).

### T-plastin favors keratinocyte migration through FAK and β6-integrin signaling

**Figure 3:**
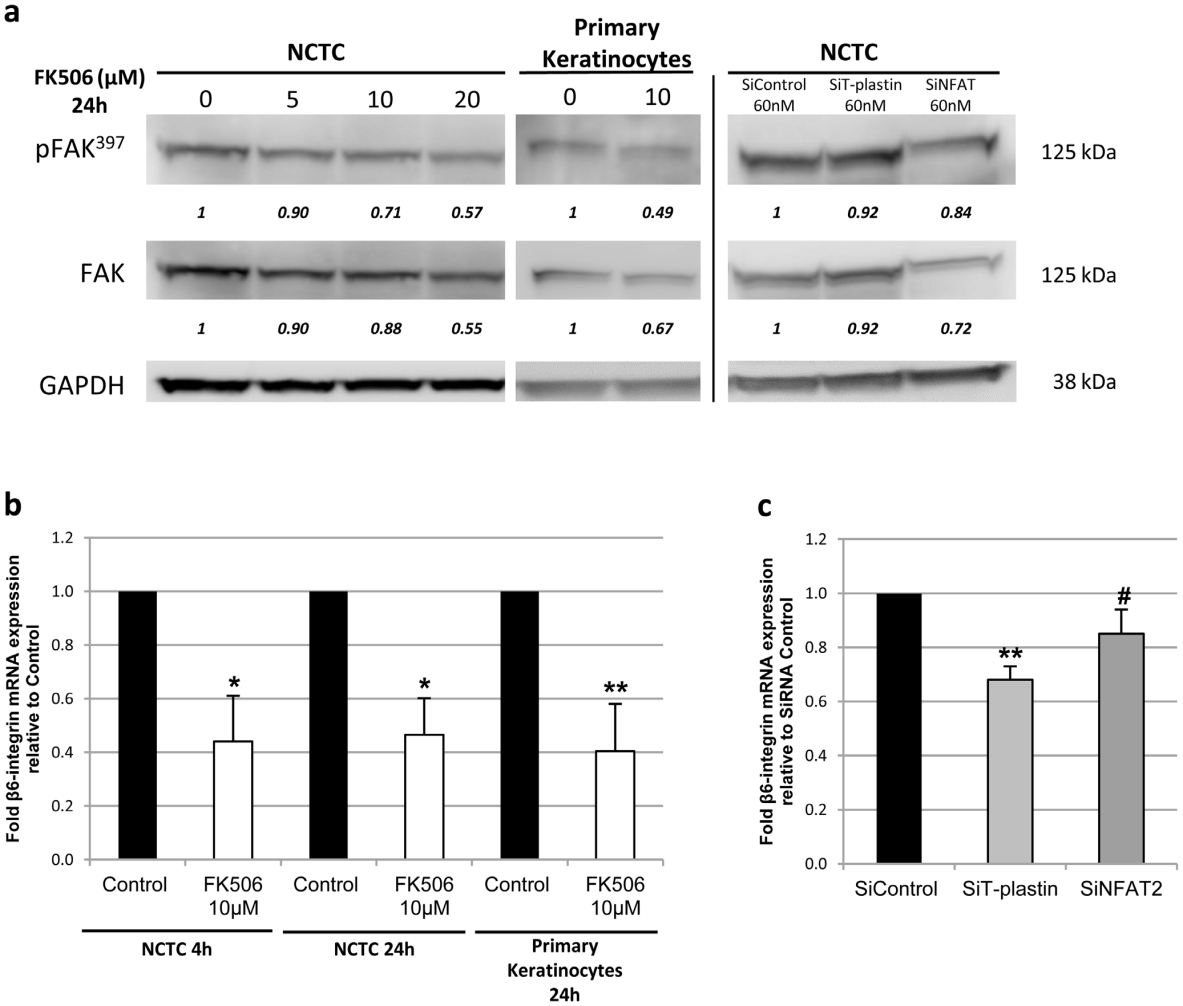
T-plastin favors keratinocyte migration through FAK and β6-integrin. (**a**) FAK protein expression was quantified by western blot analysis in NCTC (3 independent experiments) or primary keratinocytes (n = 3) after FK506 exposure for 24 h and after siRNA transfection (**b**) β6-integrin mRNA expression was quantified by quantitative RT-PCR in NCTC (3 independent experiments) or primary keratinocytes (n = 3) after FK506 treatment for 4 or 24 h and results are expressed as ratio relative to untreated control taken as 1. (**c**) β6-integrin mRNA expression was evaluated after siRNA transfection by qRT-PCR (3 independent experiments). All results are means ± SD. The values reported under each blot are the mean fold protein expression relative to controls taken as 1 after normalization by GAPDH (Image J quantification). *p<0.05, **p<0.01.

Having demonstrated that T-plastin synthesis is under the control of the activation of the calcium-dependent calcineurin/transcription factor NFAT2 pathway, we further investigated whether the role of the actin-bundling T-plastin in keratinocyte migration is linked to focal adhesion turnover through FAK pathway, a major component involved in cell migration. Indeed, the reorganization of the cell cytoskeleton is necessary for cell movement initiation and is associated with the formation and dissociation of focal adhesion contacts, as previously shown in keratinocytes [5]. Therefore, we analyzed the consequences of the decrease of T-plastin expression in keratinocytes by FK506 or NFAT2 or T-plastin siRNA treatment on the FAK protein expression. Downregulation of T-plastin expression by either FK506 or siRNA resulted in a slight but significant decrease in FAK protein autophosphorylation as well as protein expression in both NCTC and primary keratinocytes (Figure 3a). These results are in accordance with the fact that ORAI-1 is involved in keratinocyte migration. Indeed, ORAI-1 channels as well as STIM-1, by supplying intracellular calcium were shown to be necessary for focal adhesion contact formation via FAK turnover and further association with actin filaments for cell movement.

Moreover, focal adhesion kinases are known to play a prominent role in β-integrin signaling [12], indicating that β-integrins may impact cell migration. In particular, the β6-integrin, as a subunit of the α5β6 complex, was shown to be critical for keratinocyte migration [13]. In order to determine whether T-plastin expression is linked to β6-integrin, we additionally investigated β6-integrin expression after T-plastin downregulation by FK506 or transfection with specific NFAT2 or T-plastin siRNA. β6-integrin mRNA expression was only partly but significantly decreased in both NCTC and primary keratinocytes after FK506 exposure as well as in NCTC after T-plastin downregulation by siNFAT2 or siT-plastin (Figure 3b and 3c).

**Figure 4:**
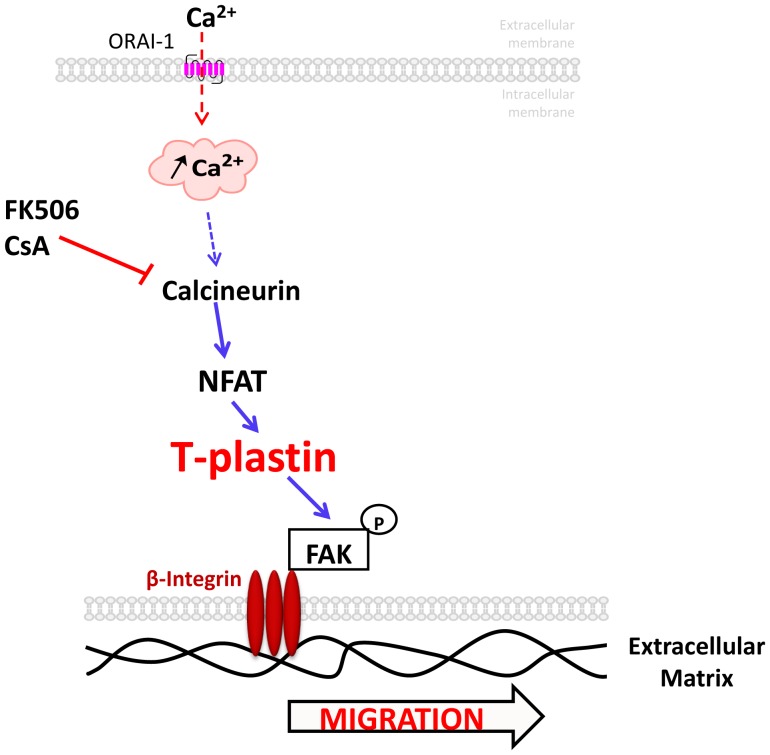
The role of T-plastin in keratinocyte migration. In response to calcium, the calcineurin/NFAT pathway activation favors T-plastin synthesis in keratinocytes. As a consequence, T-plastin through actin-bundling and stabilization of the actin network, will act on FAK expression and autophosphorylation as well as on β6-integrin expression. Altogether, this cascade will promote migration of human keratinocytes.

Altogether, these results confirm that the calcineurin/NFAT pathway is, at least partly, involved in keratinocyte motility and reveal a prominent role of T-plastin in keratinocyte migration downstream to the calcineurin/NFAT pathway through FAK turnover and β6-integrin expression, as proposed in the figure 4 that schematizes our present hypothesis.

The two well-known inhibitors of the calcineurin/NFAT pathway CspA and FK506 are largely used as immunosuppressive drugs to prevent graft rejection in organ transplantation. However, several studies highlighted an increased risk of developing skin and mucosal lesions in organ transplant patients receiving CspA or FK506 treatment [14], [15]. Under normal condition, re-epithelization of wounds requires keratinocyte migration, proliferation and differentiation. As suggested by our present data, including our results with CspA, that are quite similar to those obtained with FK506 (data not shown), the loss of migratory potential of keratinocytes under FK506 or CspA exposure linked to a decrease in T-plastin expression might contribute to the occurrence of repeated skin lesions in patients treated with these immunosuppressive calcineurin/NFAT inhibitors.
